# The economic and health impact of substandard uterotonic use for prevention of postpartum hemorrhage in three Sub-Saharan African countries: a comparative analysis

**DOI:** 10.1186/s12961-025-01322-y

**Published:** 2025-07-01

**Authors:** Petra Procter, Sara Rushwan, Yi-Fang Ashley Lee, Colleen R. Higgins, A. Metin Gülmezoglu, Lester Chinery, Sachiko Ozawa

**Affiliations:** 1 Concept Foundation, Geneva, Switzerland; 2 Division of Practice Advancement and Clinical Education, UNC Eshelman School of Pharmacy, University of North Carolina, Chapel Hill, NC United States of America; 3 Department of Maternal and Child Health, UNC Gillings School of Global Public Health, University of North Carolina, Chapel Hill, NC United States of America

**Keywords:** Postpartum haemorrhage, Uterotonics, Universal health coverage, Poor-quality medications, Low-and-middle-income countries, Sub-Saharan Africa, Health economics, Comparative analysis

## Abstract

**Background:**

Uterotonics are essential in reducing the risk of postpartum haemorrhage (PPH) and saving mothers’ lives. However, numerous quality-testing studies have found that uterotonics in many low- and middle-income countries are substandard. This study compares the economic, health, and policy implications of poor-quality uterotonics in three West African countries: Ghana, Nigeria, and Senegal. The economic impact of poor-quality uterotonics has not been previously compared.

**Methods:**

We utilized a decision-tree model to examine the implications of using substandard uterotonics (oxytocin and misoprostol) in three countries. The model simulated the place and mode of delivery, use and quality of uterotonics, risk and diagnosis of PPH and resulting economic and health outcomes. Country-specific inputs were derived from demographic and health surveys and published literature. Given large variations in population size, results were compared across 100 000 women giving birth.

**Results:**

Ghana demonstrated the greatest benefit from improvements in uterotonic quality, with US $2 million (13%) in annual cost savings and 2200 (11%) cases of PPH avoided per 100 000 women giving birth. Comparatively, annual cost savings were estimated at US $1.1 million (7%) and US $224,000 (7%) per 100 000 birthing women in Nigeria and Senegal, respectively. The yearly reduction in PPH cases per 100 000 birthing women was projected at 875 (6%) for Senegal and 944 (4%) for Nigeria. Taking varying population sizes into account, we saw that improvement in uterotonic quality could annually save US $89 million in Nigeria, US $18.8 million in Ghana and US $1.3 million in Senegal, leading to 100 000 fewer PPH cases per year overall. These simulated results were primarily driven by high proportions of substandard uterotonics and high facility use in Ghana, high numbers of home births in Nigeria and substandard misoprostol use in Senegal.

**Conclusions:**

Improving uterotonic quality would bring significant cost savings and maternal health improvements across countries. Specific policies to improve uterotonic quality and bring about the economic and health benefits may need to be tailored by country. Ensuring the quality of uterotonics is essential in improving medicine equity and would contribute towards efforts to achieve universal health coverage by ensuring that medications adequately achieve their value for money.

## Background

Postpartum haemorrhage (PPH) remains the most common direct cause of maternal mortality, accounting for one fifth of all maternal mortality and morbidity globally. PPH is defined as women facing blood loss of 500 mL or more within 24 h of birth [1]. The most important component to prevent and manage PPH is the use of uterotonics during the third stage of labour [2, 3]. However, the efficacy of uterotonics are often compromised by poor-quality medications, which pose a significant economic impact and threat to maternal health, particularly in low- and middle-income countries (LMICs) [4, 5]. Poor-quality uterotonics are often substandard, meaning they do not meet international quality standards and specifications [6].

A medicine’s quality is critical to achieving universal health coverage (UHC) and its principle of equitable access to essential healthcare without financial hardship for every person [7]. There have been efforts to investigate the relationship between UHC and medicine quality, finding that improvements in quality can be reinvested back into the health system to achieve UHC [8]. For uterotonics, which are both commonly used and have significant implications on maternal morbidity and mortality, how improvements in medicine quality can translate to UHC benefits will likely differ by country healthcare contexts.

Limited data exist in LMICs on the value of improving the quality of commonly used uterotonic agents such as oxytocin and misoprostol. Evidence from Ghana, Nigeria and Senegal indicate that there are concerns with uterotonics found in health facilities, where samples have failed quality testing owing to the amount of active pharmaceutical ingredients being below acceptable levels [4, 5, 9]. These countries, burdened with high rates of PPH and persistently high maternal mortality ratios (MMRs), confront similar challenges in ensuring access to quality-assured uterotonics. For example, Ghana reports an MMR of 234 maternal deaths per 100 000 live births [3]. Concerns raised there regarding the quality and availability of uterotonics in the market are coupled with challenges in healthcare provider training and adherence to clinical guidelines [10]. Similarly, Nigeria faces obstacles in providing access to quality-assured uterotonics, especially in rural areas where the healthcare infrastructure is varied, exacerbating supply chain challenges and medication availability [11]. Moreover, a large proportion of home births further complicates efforts to prevent and manage PPH in Nigeria [12]. While Senegal has made progress in reducing its MMR since 2000, challenges persist, particularly in rural regions where access to quality-assured uterotonics and trained healthcare providers remains limited [13].

In light of these challenges, our study aimed to demonstrate the economic and health impact of poor-quality uterotonics and their policy implications in Ghana, Nigeria and Senegal [14–16]. By highlighting the importance of safeguarding uterotonic medicine quality in LMICs to achieve UHC, this paper seeks to comparatively analyse findings across the three countries and to identify tailored interventions to mitigate the burden of PPH-related morbidity and mortality.

## Methods

We leveraged a decision-tree model to estimate country-specific impacts of substandard uterotonics in preventing PPH. The model simulated pregnant women giving birth in various places (public hospitals, primary healthcare centres, private hospitals or homes) and modes of delivery (i.e. vaginal or caesarean section). We then incorporated the use and quality of uterotonics and the risk of PPH diagnosis and modelled the associated economic and health outcomes. Information on the quality of uterotonics was extracted from the literature, where studies tested whether oxytocin and misoprostol met quality standards [5, 9, 17, 18]. Further details are described in previous studies [14, 15]. 

We utilized the most reliable country-specific data to compare countries where available. Where data were not made publicly available, insights from key informants were gathered to develop informed assumptions on uterotonic utilization. Treatment costs were obtained from sample facilities in each country, where we took mean values and utilized cost ranges in sensitivity analyses.

The primary outputs of the model were the estimates of direct costs and productivity losses, both with and without substandard uterotonics. Direct costs included out-of-pocket (OOP) costs in all three countries as well as costs incurred by the National Health Insurance Scheme (NHIS) in Ghana. Productivity losses were estimated on the basis of the number of years of maternal life lost due to PPH multiplied by the annual gross domestic product (GDP) per capita in each country, discounted at 3% to account for the time-value of money [19]. These economic outcomes were based on the health outcomes, which included numbers of PPH cases (with blood loss ≥ 500 mL), severe PPH cases (with blood loss ≥ 1000 mL) and maternal deaths due to PPH.

We ran three country models to report the baseline economic and health outcomes among 100 000 women giving birth. The benefits of ensuring the quality of oxytocin and misoprostol in health facilities were evaluated in comparison with the baseline, where uterotonic quality was determined through the chemical testing of uterotonics in these countries [5, 9, 17, 18]. We subsequently compared the simulation outcomes and policy implications across the three countries. 

## Results

Table 1 presents key model inputs by country case study. Model inputs were extracted from a recent Cochrane review, each country’s latest demographic and health surveys (DHS), the E-MOTIVE trial and other literature [12, 20, 21].

**Table 1:** Comparisons of key model inputs across three countries

Model Inputs	Senegal	Nigeria	Ghana	Source
**Demographics**				
Total population	17 316 449	213 401 323	32 833 031	[25–27]
Birth rate – per 1000 people	33	37	29	[25]
Maternal mortality ratio – per 100 000 live births	261	1047	263	
Female life expectancy at birth (years)	69	53	26.5	[25, 46, 47]
GDP per capita (USD)	1599	2066	2363	[48–50]
**Delivery locations and delivery modes**				
Urban				
Public hospital and vaginal birth (%)	10	19	42	[12, 20, 21]
Public hospital and c-section (%)	4	2	13	
Public primary health centre and vaginal birth (%)	75	16	17	
Private hospital and vaginal birth (%)	6	20	12	
Private hospital and c-section (%)	1	3	3	
Home (%)	5	39	15	
Rural				
Public hospital and vaginal birth (%)	3	8	29	[12, 20, 21]
Public hospital and c-section (%)	2	1	7	
Public primary health centre and vaginal birth (%)	62	11	26	
Private hospital and vaginal birth (%)	1	5	5	
Private hospital and c-section (%)	0	0	1	
Home (%)	31	75	31	
**Utilization of uterotonics**				
Facility				
Oxytocin (%)	34	26	34	Assumption based on KOL insights
Oxytocin and misoprostol (%)	66	52	66	
No uterotonics given (%)	0	22	0	
Home				
No uterotonics given (%)	100	100	100	
**Proportion of substandard uterotonics**				
Oxytocin				
Public (%)	5.3	80	53	[9, 17, 18]
Private (%)	4.2	72	58	
Misoprostol				
Public (%)	45.5	38	38	[5, 9, 17]
Private (%)	25	32	32	

Costs (USD)	Senegal	North Nigeria	South Nigeria	Ghana	Source
**Public hospitals**					
Vaginal birth					Collected by KOL from sample facilities
No PPH	20.44	39.30	176.85	172.24	
Mild PPH	30.97	31.44	275.10	258.37	
Severe PPH without surgery	43.16	124.45	340.60	516.73	
Severe PPH with surgery	66.76	255.45	484.70	775.10	
C-section					
No PPH	53.84	176.85	347.15	344.49	
Mild PPH	69.94	275.10	432.30	430.61	
Severe PPH	100.66	350.43	481.43	516.73	
**Primary health centres**					
Vaginal birth					
No PPH	20.44	13.10	176.85	134.57	
Mild PPH	24.97	26.06	216.15	201.85	
Severe PPH without surgery	31.40	78.60	275.10	403.70	
**Private hospitals**					
Vaginal birth					
No PPH	213.73	362.29	432.30	595.70	
Mild PPH	224.91	545.63	681.20	975.38	
Severe PPH without surgery	289.83	702.67	851.50	1621.29	
Severe PPH with surgery	1065.83	1109.00	1179.00	2879.30	
C-section					
No PPH	788.36	1205.20	1205.20	1242.60	
Mild PPH	806.03	1215.67	1441.00	1622.28	
Severe PPH	1605.83	2543.58	2849.25	1837.58	

C-*section* caesarean section, *GDP* gross domestic product, *KOL* key opinion leaders, *PPH* postpartum haemorrhage *USD* United States dollar

Key health and economic outcomes from the baseline models in Senegal, Nigeria and Ghana are presented per 100 000 women giving birth in Table 2. On the basis of the DHS data, Ghana and Senegal have similar uses of facility births at 77% and 79%, respectively, while in Nigeria, fewer than 40% of women deliver in healthcare facilities. The rest of the births take place at home, with Nigeria experiencing the highest proportion of home births, at over 60% [12, 20, 21]. Across countries, most births occur vaginally, either at home or in a facility. Nigeria has the highest rate of caesarean sections (c-sections) at around 13% of all births, whereas in Senegal and Ghana, c-sections make up only 3% of births [12, 20, 21].

**Table 2:** Key baseline health and economic outcomes in Senegal, Nigeria and Ghana annually per 100 000 delivering women

Baseline results per 100 000 birthing women	Senegal	Nigeria	Ghana
**Health outcomes**			
Percentage of facility births	77%	38%	79%
Number of vaginal facility births	73 743	35 713	66 248
Number of c-section facility births	3012	2723	12 634
Number of home births	23 245	61 564	21 118
Number of women receiving substandard uterotonics	25 266	19 795	38 553
Number of women with postpartum haemorrhage receiving substandard uterotonics	3409	3463	8415
PPH ≥ 500 mL	15 144	21 508	19 846
PPH ≥ 1000 mL	3701	4629	4925
PPH ≥ 500 mL diagnosed	3904	2230	6709
PPH ≥ 1000 mL diagnosed	1117	716	1931
Number of deaths	78	363	106
**Economic outcomes**			
Out-of-pocket costs due to PPH	US $456 521	US $3 708 245	US $8 386 499
Public facilities	US $310 615	US $968 927	US $5 005 414
Private facilities	US $145 905	US $2 739 318	US $3 381 085
NHIS costs due to PPH	–	–	US $1 272 014
Productivity losses due to PPH	US $2 955 775	US $12 391 059	US $5 902 967
**Annual total economic burden by PPH**	US $3 412 296	US $16 099 304	US $15 561 480

*C-section* caesarian section, *NHIS* National Health Insurance Scheme, *PPH* postpartum haemorrhage

On the basis of data that tested the quality of uterotonics in each country, and the proportion of women giving birth likely receiving those uterotonics at health facilities [5, 9, 17, 18], more birthing women were estimated to annually receive substandard uterotonics in Ghana (38 500 per 100 000), followed by Senegal (25 000 per 100 000) and Nigeria (19 800 per 100 000). The estimated number of women with PPH who receive substandard uterotonics was also highest in Ghana (8400 per 100 000) compared with Nigeria (3500 per 100 000) and Senegal (3400 per 100 000). This was due to high proportions of women utilizing health facilities, the frequent use of oxytocin as the single uterotonic at health facilities, and around half of oxytocin tested as being substandard (53% in public and 58% in private facilities) in Ghana.

Our model estimated that Nigeria has the highest occurrence of PPH annually at 21 500 out of 100 000 birthing women compared with nearly 20 000 out of 100 000 in Ghana and 15 000 out of 100 000 in Senegal. The high PPH burden in Nigeria is driven by many women delivering at home (75% in rural and 39% in urban areas) [12] where they face higher risks of PPH without the use of prophylactic uterotonics. Additionally, it has been reported that around 22% of women delivering at health facilities in Nigeria do not receive any prophylactic uterotonics [23]. Nigeria also has a reportedly high prevalence of poor-quality oxytocin at public (80%) and private facilities (72%), which increases the risk of birthing women experiencing PPH [17, 24].

Moreover, these PPH estimates for all three countries utilized data from a Cochrane review [24], which were aligned with the PPH burden measured through the E-MOTIVE trial [22]. Our model estimated low rates of PPH diagnosis, attributed to facilities not accurately measuring blood loss as observed in the E-MOTIVE trial [22]. The PPH burden was found to be second highest in Ghana due to many c-sections and higher prevalence of substandard oxytocin in Ghana (53–58%) compared with Senegal (4.2–5.3%) [5, 9, 18, 20, 21].

Nigeria was estimated to have the highest number of yearly deaths due to PPH (around 360 per 100 000 women giving birth), followed by Ghana (approximately 100 per 100 000) and Senegal (around 80 per 100 000). These estimated deaths were based on each country’s MMR and the number of PPH cases. The high burden of PPH deaths resulted in high annual productivity losses (US $12 million per 100 000 birthing women) in Nigeria.

Annual costs associated with PPH in Nigeria and Ghana were estimated at around US $16 million per 100 000 women giving birth, whereas in Senegal, they were estimated at US $3 million per 100 000 women giving birth. Ghana showed the highest OOP costs for PPH, at US $8.4 million per 100 000 birthing women (US $5 million in public facilities and US $3.4 million in private facilities), reflecting the high proportion of women utilizing health facilities for delivery. Senegal had the lowest OOP costs at US $457 000 per 100 000 births, with US $311 000 incurred in public facilities and US $146 000 in private facilities. The NHIS in Ghana annually bears some PPH costs (US $1.2 million) associated with facility care. A similar NHIS structure does not currently exist in Senegal or Nigeria.

While utilization of healthcare services and the PPH burden affect OOP expenses, unit costs also varied within and across countries. The unit costs of healthcare varied the most within Nigeria, where the mean cost of treating a mild PPH case at a primary health center was estimated at US $26 in the North and US $216 in the South. Across countries, costs to treat PPH at a public hospital were the highest in Ghana, spanning from US $258 to US $775, compared with US $31–485 in Nigeria and US $31–101 in Senegal.

Table 3 compares the key benefits of improving the quality of uterotonics in reducing PPH cases and accruing annual cost savings per 100 000 birthing mothers across Senegal, Nigeria and Ghana. Of the three countries, Ghana demonstrated the most substantial benefit from improvements in uterotonic quality, with 2200 avoided cases of PPH (11%) and US $2 million in savings (13%) per 100 000 birthing women annually. Comparatively, the annual reduction in PPH cases per 100 000 birthing women was projected at 875 for Senegal and 944 for Nigeria. Annual cost savings were estimated at US $1.1 million (7%) and US $224 000 (7%) per 100 000 birthing women in Nigeria and Senegal, respectively. Breaking down the cost savings further, Ghana showed the largest reduction, with OOP public facility costs decreasing by US $689 000 (14%), OOP private facility costs by US $523 000 (15%) and productivity loss savings amounting to US $653 000 (11%) per 100 000 birthing women. Nigeria followed, with OOP public facility savings of US $126, 000 (13%), OOP private facility savings of US $377 000 (14%) and productivity loss savings of US $628 000 (5%) per 100 000 birthing women. Meanwhile, Senegal’s cost savings were lower owing to relatively lower healthcare costs, with reductions of US $28 000 (9%) in OOP public facility expenses, US $8000 (6%) in OOP private facility expenses and US $188 000 (6%) in productivity losses per 100 000 birthing women.

**Table 3:** Annual health and economic benefit by ensuring the quality of uterotonics in Senegal, Nigeria and Ghana per 100 000 women giving birth

Benefits of using quality uterotonics	Annual PPH case reduction (IQR)		Annual cost savings (IQR)			
			OOP public facilities	OOP private facilities	Productivity losses	Total
Senegal	Estimates	875 (724–1018)	US $27,935 (14 126–33 044)	US $8097 (−640 to 16 792)	US $187 584 (−113 435 to 491 552)	US $223 616 (−86 586 to 522 662)
	%	6%	9%	6%	6%	7%
Nigeria	Estimates	944 (794–1097)	US $125 757 (95 723–152 313)	US $377 314 (124 994–468 762)	US $628 443 (34 127–1 228 561)	US $1 131 514 (528 038–1 754 916)
	%	4%	13%	14%	5%	7%
Ghana	Estimates	2179 (1909–2415)	US $688 648 (582 584–779 636)	US $523 210 (426 426–615 720)	US $652 805 (167 687–1 173 808)	US $2 040 510 (1 463 815–2 588 662)
	%	11%	14%	15%	11%	13%

*IQR* interquartile range, *OOP* out-of-pocket, *PPH* postpartum haemorrhage

Our model projected the greatest impact from ensuring uterotonic quality in Ghana owing to high proportions of women birthing at facilities (79%) coupled with high proportions of uterotonics being substandard in Ghana (53–58% of oxytocin and 32–38% of misoprostol). While Nigeria has worse prevalence of substandard oxytocin (72–80%) on the basis of available quality testing results, facility use is low (38%) which reduced the impact of improving the quality of uterotonics used in facility births. Senegal has a problem with substandard misoprostol (25–46%); however, the cost of healthcare is lower, resulting in less cost savings from uterotonic quality improvements.

Population size varies significantly between the three countries, with over 213 million people in Nigeria, 33 million in Ghana and 17 million in Senegal [25–27]. At the population level, our model estimated that improving the quality of uterotonics could avert nearly 75 000 PPH cases and save US $89 million annually in Nigeria including over $40 million in OOP costs [15]. In Ghana, improving the quality of uterotonics could reduce 20 000 PPH cases and contribute to US $18.8 million in cost savings annually [14]. In Senegal, our model showed that ensuring that all uterotonics are quality-assured could avert 5000 PPH cases and save US $1.3 million in costs annually [16].

## Discussion

This study utilized a modelling approach to compare the impact of improving uterotonic quality for PPH prevention in Ghana, Nigeria and Senegal. The findings highlighted the financial and health benefits of ensuring quality uterotonics across all three countries. Among them, Ghana showed the most substantial benefit from improvements in uterotonic quality, with US $2 million in savings (13%) and 2200 avoided cases of PPH (11%) per 100 000 women giving births. In comparison, annual cost savings were estimated at US $1.1 million in Nigeria and US $224 000 in Senegal per 100 000 birthing women. Both Nigeria and Senegal were projected to see annual reductions of 944 and 875 PPH cases per 100 000 birthing women, respectively.

The economic impact of poor-quality uterotonics has not been compared across countries previously. Our models showed that improving the quality of uterotonics could result in significant annual savings at the population level, amounting to US $89 million in Nigeria, US $18.8 million in Ghana and US $1.3 million in Senegal. Additionally, these improvements could also lead to a reduction in 100 000 PPH cases annually. These simulated results were primarily influenced by factors such as the prevalence of substandard uterotonics, high facility utilization in Ghana, low facility-based births in Nigeria and the use of inadequate quality misoprostol in Senegal.

In Nigeria, where the population is significantly larger, the importance of improving uterotonic quality should be underscored. The latest available data reported an MMR of 993 maternal deaths per 100 000 live births [3], indicating a critical need for intervention. Our model highlights that improvements in uterotonic quality could lower maternal mortality in Nigeria; however, the benefits would become even more apparent if access to uterotonics also increased either through growth in institutional delivery or the use of quality uterotonics at home. There has only been a slight increase in the reported percentage of deliveries occurring at health facilities in Nigeria over the past decade – from 35% in 2008 and 36% in 2013 to 39% in 2018 [12]. Furthermore, there are disparities between urban and rural birth settings in Nigeria, with 61% of urban births being delivered in a health facility compared with only 26% of rural births [12]. Increasing the use of quality uterotonics will look different in each setting. Increasing the number of mothers who utilize healthcare facilities for childbirth could also be effective in preventing other causes of maternal deaths [28]. Furthermore, making improvements to the quality of uterotonics at facilities in Nigeria could be a way to encourage more mothers to make use of health facility care during labour and delivery.

The implementation of the NHIS in Ghana in 2005, followed by the incorporation of free maternal healthcare into the insurance scheme in 2008, have significantly enhanced healthcare utilization in Ghana [29, 30]. Under these policies, all pregnant women are eligible for free registration with the NHIS, granting them access to services throughout their pregnancy, during childbirth and for 3 months postpartum. This policy is an effort towards Ghana’s commitment to achieving the Sustainable Development Goals, particularly in reducing maternal and child mortality rates and advancing UHC [29]. Access to quality uterotonics plays a pivotal role in realizing the objectives of this free maternal health policy, because quality uterotonics are necessary to better health outcomes for mothers without burdening them with additional costs. To achieve the desired improvement in maternal health outcomes, this study has shown that uterotonic quality at both public and private facilities must be assured.

Ghana and Senegal have relatively similar proportions of women delivering at health facilities. In Senegal, facility births reached nearly 80% in 2016 [20]. However, most births in Senegal occur in lower-level facilities with limited resources to provide quality care [31]. A post-market surveillance study indicated that a high proportion of misoprostol sampled in Senegal was of substandard quality [9]. Even though misoprostol does not require cold chain or storage at refrigerated temperatures, it has been shown to deteriorate quickly under poor storage conditions resulting from exposure to moisture [32]. Whereas Ghana and Nigeria may need to focus efforts in quality improvements on cold chain storage to protect the quality of oxytocin, Senegal may benefit from focusing on good shipment and storage practices for misoprostol.

Policy-focused interventions are required to address the issue of poor-quality uterotonics, which may include procurement procedures and supply chain management, strengthening regulatory oversight and promoting adherence to clinical guidelines and good medicine storage awareness/behaviours [23, 33–35]. This requires an awareness of the problem followed by financial and political commitment to implement processes that would ensure medicine quality. In LMICs where the quality of uterotonics cannot be easily guaranteed, one option is to invest in heat-stable, quality-assured uterotonics such as heat-stable carbetocin. This could improve the efficiency of public health government spending by reducing the number of PPH cases, as recommended by WHO [37] and demonstrated in a cost-effectiveness/budget-impact model in India and Uganda [38, 39]. Future research could examine PPH-related cost-effectiveness analyses to assess the impact of incremental costs of ensuring uterotonic quality over time on health system resource utilization and health outcomes across settings [38, 39].

Furthermore, to enhance the diagnosis and treatment of PPH as well as ensure the quality of uterotonics in shipping, storage and handling, it is important to support initiatives aimed at improving the training of healthcare providers, particularly in rural areas [36]. Investment in such training coupled with the provision of quality-assured uterotonics can lead to reduced productivity losses and improved maternal health outcomes. Ultimately, a multisectoral effort at the global, national and subnational levels is necessary to guarantee universal accessibility of quality-assured uterotonics without financial hardship.

It is important to remember that the economic burden associated with PPH extends beyond the direct costs of healthcare and productivity losses that are estimated in this study. Maternal mortality and morbidity can have significant negative effects on economies, communities and families. These effects may include financial instability and lower economic production, loss of education or lower educational achievement of children, increased childhood mortality and higher poverty [34, 40]. Therefore, the potential benefits of ensuring quality uterotonic use are likely to be even greater than the estimates presented in our models.

Our findings resonate with previous studies that have identified inadequate access to quality healthcare facilities and life-saving medicines as critical barriers to reducing maternal mortality in LMICs [41, 42]. However, there are limitations, including the quality and completeness of the underlying data sources, which are affected by scarce literature. In addition, key assumptions made in modelling may impact the relevance of the estimations. One consideration is the variation in PPH definitions. While we applied a widely used definition of PPH (≥ 500 mL blood loss) across all birthing modes [43, 44], some guidelines define PPH after caesarean delivery as blood loss exceeding 1000 mL [45]. These definitional differences could impact our estimates. However, using a consistent threshold across delivery modes allows for better comparability and broader public health insights. Furthermore, the generalizability of the results may be limited by contextual factors that differ across the three countries, such as the variations of the healthcare system dynamics and varying impacts of social determinants of health on populations. Further research should generate evidence on the value of ensuring medicine quality across populations and settings.

## Conclusions

Improving the quality of uterotonics has the potential to bring about significant cost savings and maternal health outcome improvements across West African countries. The strategies and policies to achieve these financial and health improvements need to be tailored to the specific contexts and needs of each country. With the right strategies and policies in place that ensure a quality uterotonic is used to prevent PPH in each delivery, families and governments will realize a greater value for their money and attain stronger stewardship of scarce financial resources.

## Abbreviations

DHS: Demographic and health survey
GDP: Gross domestic product
LMICs: Low-and-middle income countries
MMR: Maternal mortality ratio
NHIS: National health insurance scheme
OOP: Out of pocket
PPH: Postpartum haemorrhage
UHC: Universal health coverage
WHO: World Health Organization

## Declarations

### Availability of data and materials

No datasets were generated or analysed during the current study.
